# Effect of High-Intensity Interval Training, Moderate Continuous Training, or Guideline-Based Physical Activity on Peak Oxygen Uptake and Myocardial Fibrosis in Patients With Myocardial Infarction: Protocol for a Randomized Controlled Trial

**DOI:** 10.3389/fcvm.2022.860071

**Published:** 2022-04-11

**Authors:** Xiaohe Shi, Xianyuan Chen, Xinfan Qiu, Wei Luo, Xinyi Luo, Hui Liu, Qingshan Geng, Huan Ma, Ling Xue, Lan Guo

**Affiliations:** ^1^Guangdong Cardiovascular Institute, Guangdong Provincial People's Hospital, Guangdong Academy of Medical Sciences, Guangzhou, China; ^2^Guangdong Provincial People's Hospital, Guangdong Academy of Medical Sciences, Guangzhou, China

**Keywords:** myocardial infarction, high-intensity interval training, moderate continuous training, peak oxygen uptake, myocardial fibrosis

## Abstract

**Introduction:**

High-intensity interval training (HIIT) is an emerging method of cardiac rehabilitation, which is more and more popular in recent years. Research into the effect of HIIT on peak oxygen uptake (VO_2_ peak) and myocardial fibrosis among patients with myocardial infarction (MI) is lacking. Here, we describe the rationale along with the protocol for a clinical trial to test the following hypotheses: (1) compared with the control group, VO_2_ peak will be increased in both the moderate-intensity continuous training (MICT) and HIIT groups and (2) compared with the control group, myocardial fibrosis due to MI will be improved by HIIT and MICT.

**Methods and Analysis:**

This is a single-center, randomized controlled clinical trial. In total, 180 patients with MI are to be recruited for this study. VO_2_ peak will be tested by cardiopulmonary exercise testing (CPET) and myocardial fibrosis will be evaluated by cardiac MR. A variety of blood and psychometric tests and also the peripheral arterial tonometry, reactive hyperemia index for microvascular endothelial function, and microvascular blockage or digital vasomotor response are included.

**Ethics and Dissemination:**

The ethics committee of the Guangdong Provincial People's Hospital has authorized this mechanistic clinical research. Peer-reviewed articles and conference presentations will be used to disseminate the findings.

**Trial Registration Number:**

NCT04863677.

## Introduction

More than 7 million people suffer from acute myocardial infarction (AMI) worldwide each year (1). And in the past decades, more and more numbers of research have shown significant reductions in AMI mortality with more effective treatment (1, 2). Despite the decreasing mortality after the first myocardial infarction (MI), 20% of MI survivors have a second cardiovascular event in the first year after MI onset (3). More sufficient and effective post-MI rehabilitation methods are in need to reduce the occurrence of adverse events.

Numerous investigations have documented that cardiac rehabilitation (CR) will prolong the survival time of patients with MI, decrease the risk of readmission and enhance the quality of life (4–6). Multidisciplinary exercise-based CR is recommended for individuals with coronary artery disease for maintaining a healthy lifestyle and regulating risk factors to reduce all-cause along with cardiovascular mortality, and morbidity and improve health-related quality of life (Class I, Level A) (7–10). India's study enrolled 3,959 patients with acute MI. The subjects were assigned at random into either the Yoga-CR program (*n* = 1,970) or improved conventional treatment with educational counseling (*n* = 1,989). The researchers found the Yoga-CR group had a greater return to preinfarct (11), short-term cardiac rehabilitation after MI did not increase survival, psychological wellbeing, or health-linked quality of life in the long run, according to a randomized controlled experiment (Rehabilitation After Myocardial Infarction Trial) by Barry A (12). Given the inconsistent results of previous studies, it is necessary to reassess the effects of different forms of exercise training for patients with MI. Peak oxygen uptake (VO_2_peak) is the gold standard for determining functional limitations and the integrative physiology of the circulatory, respiratory, muscular, cellular, and oxidative systems (13, 14). Exercise therapy, as the core of CR, improves VO_2_peak. With the increment of VO_2_ peak by 1 ml·kg^−1^·min^−1^, death risk in individuals with coronary heart disease (CHD) decreases by 15% (9). VO_2_ peak is, likewise, a remarkable individual predictor of mortality and morbidity (15, 16). The purpose of this study is to clarify the effect of two different training methods on VO_2_ peak in patients with MI. High-intensity interval training (HIIT) is defined by repeated high-intensity movements, interspersed with low-intensity recovery periods. Moderate-intensity continuous training (MICT) improves endurance mainly, demonstrating an inferior effect on VO_2_ peak compared with HIIT (17). Despite evidence that HIIT raises VO_2_ peak more than MICT, the reasons behind this advantage remain unknown. Given prior data, it is unknown if HIIT results in a greater increase in cardiac output than MICT. In comparison to MICT, HIIT seems to stimulate more genes involved in the control of mitochondrial biogenesis and oxidative enzymes in skeletal muscle (18). In addition, HIIT was shown to decrease the left ventricle's peak apical rotation and torsion (19). The energy required for myocardial contraction to meet daily workload may be reduced as a result of the reduction in peak left ventricular torsion generated by HIIT. In addition, HIIT decreased the time required to achieve the peak value of the left ventricle (19). Dun et al. (20) discovered that supervised HIIT may result in a substantial reduction in total fat mass and abdominal fat percentage, and an improved lipid profile, in patients undergoing CR who had MI. They also indicated that supervised HIIT, compared to MICT, resulted in remarkable improvements in metabolic syndrome and body composition in individuals with MI (21). It is worth further investigation to compare the effects of the two different exercise training programs in patients with MI.

Following MI, cardiomyocyte necrosis initiates an inflammatory cascade that clears the wound of dead cells, induces collagen production, activates myocardial fiber cells, and retains the ventricle's structural integrity (22). When myocardial fibrosis continues to develop, myocardial contractility and coronary flow reserve both decrease eventually leading to malignant arrhythmia and cardiac death (23). Thus, preventing and reversing myocardial fibrosis is a critical component of the postinfarction approach. Alemasi et al. (24) hypothesized that exercise training protects the myocardium against isoproterenol-induced fibrosis and diastolic dysfunction by suppressing cytokines in the Hedgehog and RAP1 pathways. However, another study used cardiac magnetic resonance (CMR) to detect myocardial fibrosis in asymptomatic triathlon athletes, showed that among 54 male triathlon athletes, there are 9 players (17%) having myocardial fibrosis, which shows that excessive physical training can lead to the occurrence of myocardial fibrosis (25).

Cardiac magnetic resonance (CMR) is a non-invasive method for evaluating CHD. It is the gold standard technique for assessing myocardial fibrosis and cardiac function, and scarred myocardium in patients with MI (26). Edema, an important character of myocardial impairment in the process of acute MI (27), will affect CMR assessment of myocardial fibrosis. There was no change in swelling for the first week; however, after 15 days the amount of edema dropped dramatically (27). A larger peak in myofibroblast surface area seven days after MI was seen in a rat experiment in which researchers discovered that myofibroblasts were present for up to 14 days in a ligated MI model (28). According to the relation in ages of laboratory rats with humans (29), in this study, we will enroll patients 6 weeks to 3 months after MI, evaluate myocardial fibrosis by CMR at baseline and 3 months after exercise training, to explore the effect of different exercise program on myocardial fibrosis.

The main objective of the study is to explore the effect of HIIT, MICT, and guideline-based physical activity on VO_2_peak and myocardial fibrosis in patients with MI. The secondary objective is to assess the effect of HIIT, MICT, and guideline-based physical activity on endothelial function, body mass index (BMI), arrhythmias, and inflammatory markers.

## Methods and Analysis

### Study Design

This study is now being carried out at the Guangdong Provincial People's Hospital in Guangzhou, Guangdong, China. The research methodology and procedures (No. GDREC2020208H(R1)) were approved by the ethics committee of the Guangdong Provincial People's Hospital.

The HIIT on MI study is an ongoing randomized controlled trial (RCT), followed up with a 1-year observing period. The first patient was recruited on April 2021. This study is to be completed by March 2024. After completing a written permission form, patients are evaluated to see whether they are eligible.

**Figure 1:**
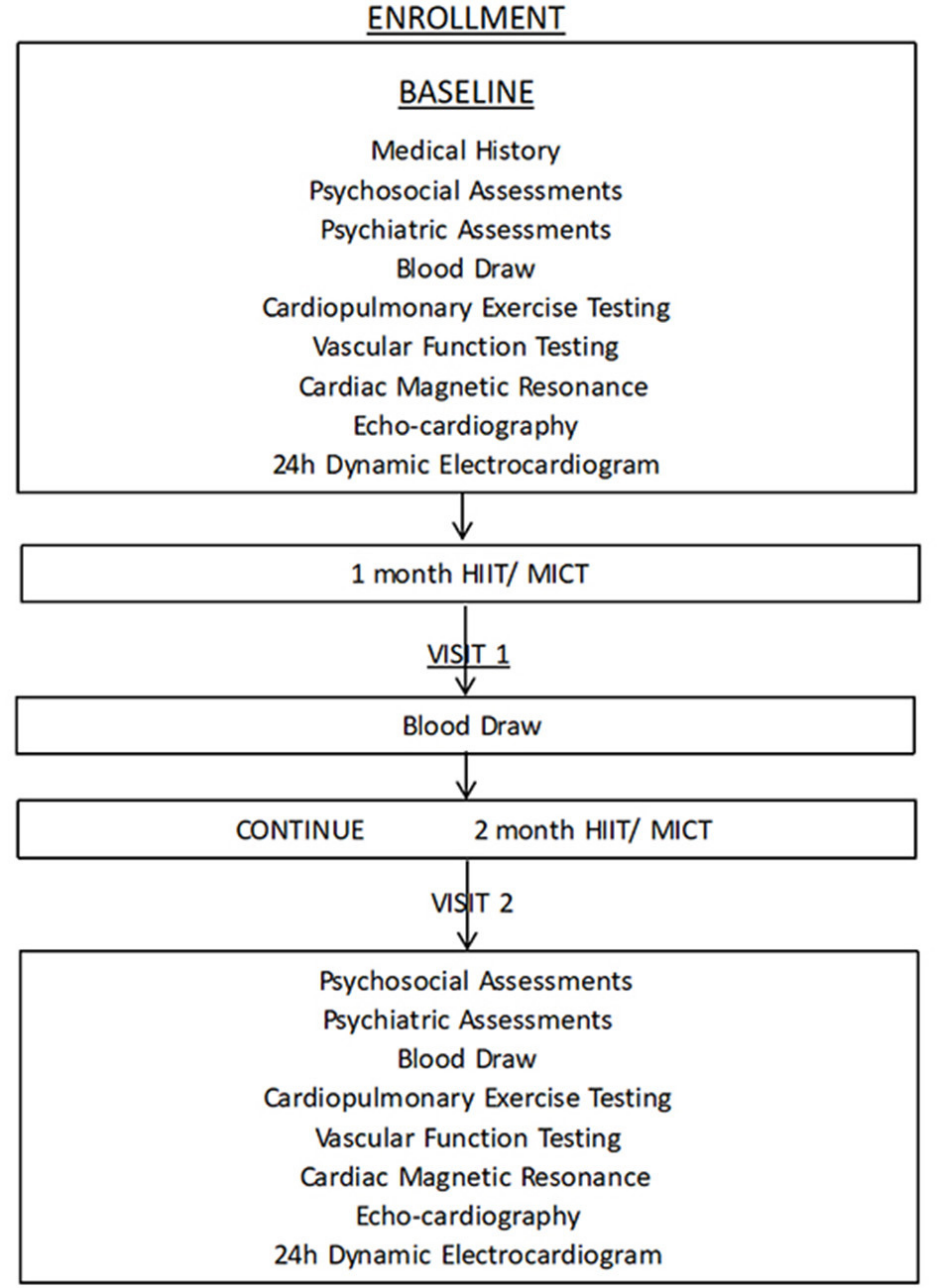
The process of study.

A 12-week cardiac rehabilitation training program (HIIT, MICT, and guideline-based physical activity) will be studied to determine the impact of HIIT and MICT on VO_2_peak, cardiac fibrosis, endothelial function, microvascular obstruction, arrhythmia, body fat, inflammation, and psychological effects on participants. The schematic design of the study is shown in Figure 1.

### Inclusion Criteria

(1) Age range: 18 to 75 years.

(2) Individuals with AMI who were treated with revascularization 6–12 weeks after the beginning of the AMI and harbored a New York Heart Association functional classification (NYHA) Class I–III; (AMI verified *via* the Fourth Universal Definition of Myocardial Infarction). The term “acute myocardial infarction” must be utilized when there is medical evidence of acute myocardial ischemia along with a detection of a fall and/or rise in cTn values with manifestations of myocardial ischemia; new alterations in ischemic ECG; developing pathological Q waves; images exhibiting prove of new loss of viable myocardium or new abnormality of regional wall motion in a pattern congruent with an ischemic etiology; and determination of a coronary (not for type 2 or 3 MIs).

(3) Able and willing to finish the CR program independently, as determined by the informed consent form.

(4) Complete cardiopulmonary exercise testing (CPET) during enrollment or within 1 month of receiving the CPET result.

### Exclusion Criteria

(1) onduct high-intensity exercise regularly.

(2) Currently enrolling in any other clinical trials.

(3) Have any other significant health condition in the preceding year (e.g., severe valvular disease, congenital heart disease, recurrent ventricular arrhythmias, NYHA Class IV heart failure, atrial fibrillation, cancers, and end-stage renal or liver disease).

(4) Dementia; infirmity; or inability to participate in sports (musculoskeletal limitations, namely, injuries, frailty, and weakness).

(5) Acute liver failure with severe complications.

(6) Acute renal failure with severe obstructive nephropathy.

(7) Irregular vital signs: heart rate at rest <40 or > 120 bpm, or blood pressure: systolic blood pressure > 200 mm Hg, diastolic pressure > 110 mm Hg, *T* ≥ 38.5 or ≤ 36°C, SpO_2_ ≤ 90%.

(8) Inspectors at the site determine that the patient will be unable to finish the trial and/or attend follow-up.

If the patient has ischemic performance during exercise, such as chest pain and chest tightness, immediately stop exercise, and re-evaluate. According to the assessment of professional cardiologists, if surgery is needed, surgical treatment will be carried out, and such patients will withdraw from the study. If it can be relieved after rest, a new exercise plan will be re-established.

### Sample Estimation

Based on the degree of myocardial fibrosis at the primary endpoint, the sample size was estimated using G Power3.1.9.4, one-way ANOVA, and *a priori* followed by an *F* test was performed. Since there is no previous reference for similar research data, the effect size of the observable indicator in this study is set to be 0.25. To achieve more than 80% power and a 2-sided critical threshold of 0.05, each arm must contain a minimum of 53 patients. Estimating a 10% loss to follow-up, the recruitment goal is 60 patients per arm (*n* = 180).

As for VO_2_ peak, based on our pre-experiment, the mean increase of peakVO_2_ in control, MICT, and HIIT groups after exercise training is −0.18, 2.86, and −0.36 ml/kg/min, and the standard deviation of the difference between groups is 2.92, 3.38, and 2.74. By one-way ANOVA *F*-tests, sample sizes of 18, 18, and 18 are obtained from the 3 groups whose means are compared. The total sample of 54 subjects achieves 80% power to detect differences among the means of peakVO_2_ using an *F*-test with a 0.05 significance level. Estimating a 10% loss to follow-up, the recruitment goal is 20 patients per arm (*n* = 60).

Taking two primary endpoints together, the recruitment goal is 60 subjects per arm.

### Randomization

Patients who are both the eligible and willing are randomized in a 1:1:1 ratio (block randomization). The computer generated a random number table with values ranging from 1 to 180 (including shedding instances). Each participant in each group will be allocated a case number in accordance with the sequence of grouping, and each case number will relate to group 1, 2, or 3 according to the random number.

### Study Procedures

Supervised exercise training will be performed 3 times per week at the CR center in the Guangdong Provincial People's Hospital. All patients are assigned at random in a 1:1:1 ratio to one of three groups: HIIT (group 1), MICT (group 2), or control group (group 3). This program consists of 36 CR sessions and typically lasts 12 weeks (3 sessions per week).

All patients receive drug treatment after MI, routine health education, and postoperative medication guidance according to the guidelines. The control group receives no extra rehabilitation or physical activity. Patients in the groups 1 and 2 perform MICT during the first week of CR, after which, they will enter the corresponding group according to the random number of the entry period.

**Figure 2:**
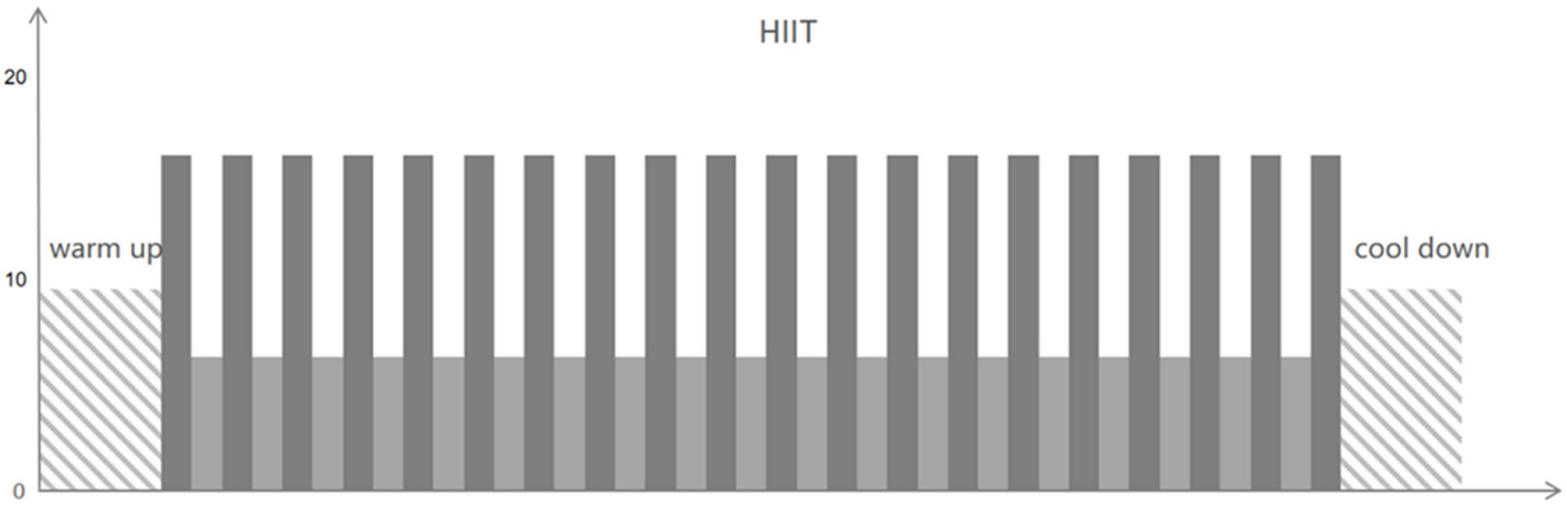
High-intensity interval training (HIIT).

**Figure 3:**
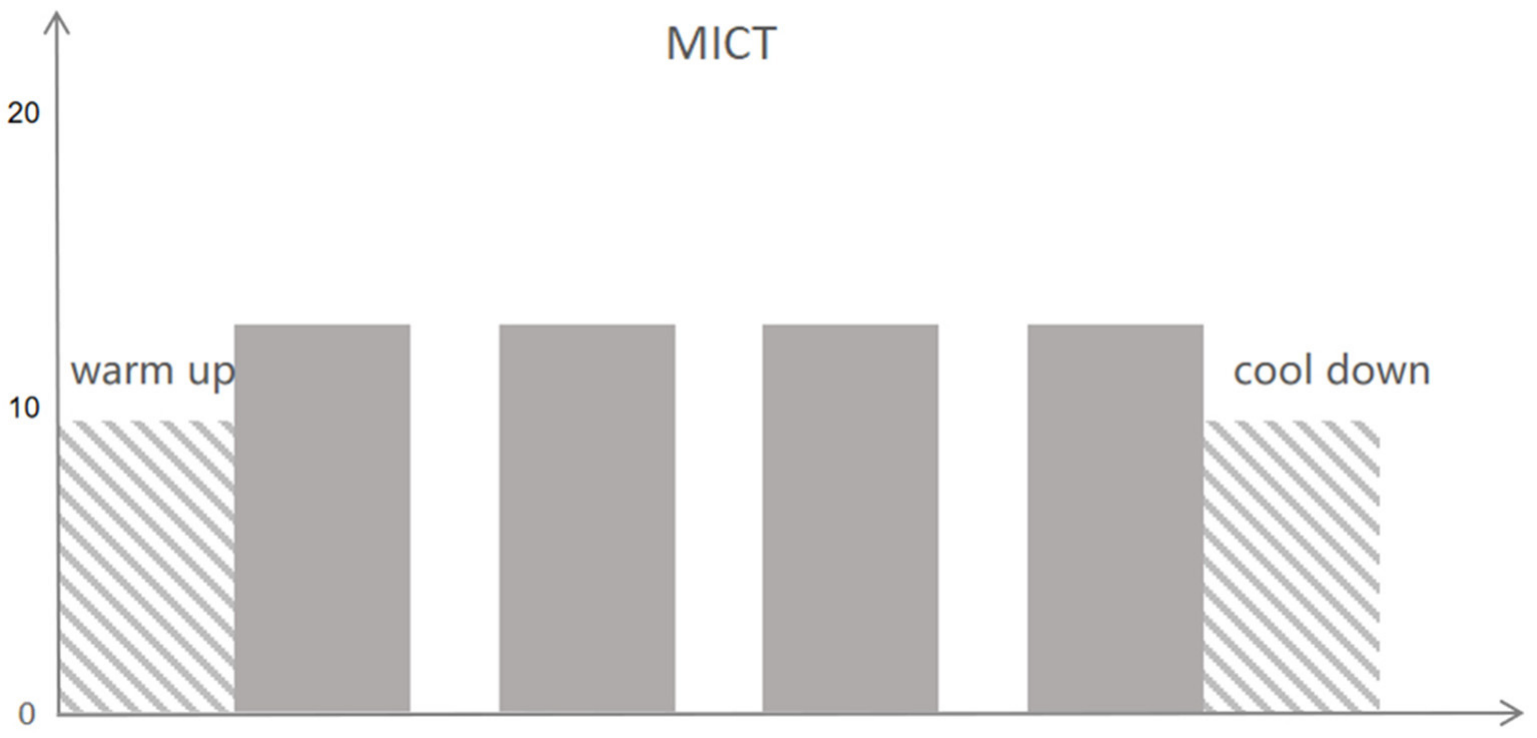
Moderate-intensity continuous training (MICT).

Exercise intensity is personalized based on the results of CPET. During CPET, patients try their best to obtain peakVO_2_ data. According to the percentage of peakVO_2_ to design exercise training programs: high strength refers to 85 to 100% peak VO_2_ and 85% peak VO_2_ is higher than the anaerobic threshold (AT); medium intensity: 40 to 60% peak VO_2_, and 60% peak VO_2_ is less than AT; and low intensity means 20% peak VO_2_. The exercise intensity also could be micro-adjusted according to the rating of perceived exertion (RPE) during the actual cardiac rehabilitation exercise. Short bursts of high-intensity exercise are alternated with intervals of low-intensity activity in HIIT programs (active recovery). HIIT constitutes twenty intervals of high effort (30–60 s at an RPE (Borg scale of 6–20) of 15–17) along with a low intensity (1 min at an RPE (Borg scale of 6–20) of 10 or completely rest). It takes about 40–50 min to complete the entire training cycle (Figure 2). Patients in the MICT group exercise for 40–50 min at an RPE of 12 to 14 (four sets of 5–8 min each, with a 2-min rest in between) (Figure 3). HIIT and MICT are only done under supervision once a week on a non-consecutive 3-day schedule. A professional CR team also keeps an eye on all of the patients. Clients may also participate in 3 days of home-based exercises guided by a sports wristband after being evaluated by therapists and physicians.

Supervised exercise, counseling, catering management, counseling on cessation of smoking, stress management, and patient education, along with psychological treatment are all part of the CR program. A typical 36-session course of CR lasts around 12 weeks (three sessions per week).

According to the 2020 European Society of Cardiology (ESC) guidelines for the management of acute coronary syndromes and the 2019 Chinese guideline of acute MI. A loading dose of 150–300 mg (or 75–250 mg) of aspirin is advised for all patients without adverse effects, and a maintenance dose of 75–100 mg od for long-term treatment [Class I, Level A, (I, A)]. Unless there are adverse events or a considerable risk of bleeding, aspirin should be taken for at least a year (I, A). In all the patients with MI, statins are indicated. Low-density lipoprotein cholesterol contents must be reduced by at least 50% from pretreatment levels, or below 1.4 mmol/l (55 mg/dl) (I, A). Unless contraindicated, angiotensin-converting enzyme (ACE) repressors [or angiotensin receptor blockers (ARBs)] in cases of intolerance to ACE repressors are recommended to minimize all-cause, and cardiovascular mortality along with morbidity in individuals with heart failure, diabetes, or chronic kidney disease (for instance, severe renal impairment, hyperkalemia, etc.). For a long period, all patients with MI should be treated with ACE inhibitors or ARBs (IIa, A). Beta-blockers are advised for those who have heart failure [left ventricular ejection fraction (LVEF) 40%] or systolic left ventricular dysfunction (I, A). In those who have had a prior MI, long-term oral beta-blocker medication should be explored to minimize all-cause along with cardiovascular mortality, and cardiovascular morbidity (IIa, B) (10).

### Cardiopulmonary Exercise Testing

Before CR exercise training, a CPET should be conducted by bicycle. We select the Ramp protocol of 10–20 W/min and conduct the symptom-constrained maximum load exercise test. Subjects need to maintain a power bike speed of (60 ± 5) r/min, complete exercise tests in 8–12 min until symptoms limit. Patients may interrupt the test if they experience dyspnea, leg weariness, symptoms (such as chest pain and dizziness), or any other unbearable discomfort. Continuous electrocardiography, gas exchange, and blood pressure along with SpO_2_ measurements will be obtained during the baseline rest, exercise, and recovery periods. A face mask is used to measure expiratory flow, VO_2_, and minute ventilatory equivalent (VE) (ventilation) along with VCO_2_ (carbon dioxide production) breath by breath. Heart rate and SpO_2_ are assessed *via* pulse-by-pulse filtering fingertip oximeters. VO_2_ peak is the highest value of oxygen uptake obtained during the exercise. The VE/VCO_2_ (ventilatory equivalent to carbon dioxide) slope is determined *via* plotting the linear regression slopes of VE and VCO_2_ from the first second of exercise to the respiratory compensation point, which occurs when acidemia-triggered ventilation along with end-tidal partial pressure of carbon dioxide (P_ET_CO_2_) start to decline. The AT is the VO_2_ level at which the VE/VCO_2_ ratio begins to drop or remains constant while the ventilatory equivalent to oxygen (VE/VO_2_) ratio continues to escalate. The ΔSpO_2_ is calculated by subtracting the peak SpO_2_ from the rest SpO_2_. The volume of oxygen at the ventilatory threshold (VT) is the beginning point for ventilation to grow at a quicker pace than oxygen consumption. The highest power output generated by the participant is known as the workload peak. The level of power output generated at VT is known as workload @VT (30–34).

### Cardiac MR

All patients in each group were chosen to have their cardiac fibrosis assessed using CMR at two different times: before the trial began and after the third month of follow-up. CMR is the gold standard for non-invasive cardiac tissue characterization. CMR allows late gadolinium enhancement techniques to characterize focal alternative fibrosis. CMR may also be employed to evaluate diffuse myocardial fibrosis by quantifying the extracellular volume fraction *via* native along with postcontrast T1 mapping approaches adjusted to the patient's hematocrit. In this study, we evaluate myocardial fibrosis in patients with MI by CMR at 6 weeks and re-examined CMR 3 months later.

### 24 h Dynamic Electrocardiogram and Echocardiography

All the patients have dynamic electrocardiogram and echocardiography recorded at the start of the research and the third month of follow-up. Without knowing the precise clinical situations, the data were independently assessed by two qualified doctors using the double-blind procedure.

### Blood Collection and Tests

Several blood samples will be obtained: before the start of the study, the first month of follow-up, and the third month of follow-up. We will detect blood routine, liver function, renal function, lipid metabolism, interleukin-6 (IL-6), platelet aggregation rate, n-terminal pro-brain natriuretic peptide (NT-proBNP), hypersensitive troponin T, stromelysin-2 (ST-2), and metabolomics measurements. We will evaluate the changes of pathogenesis from the aspects of the inflammatory mechanism, the humoral immunity index, and metabolomics measurements.

### Vascular Assessment

The Endo-PAT2000 (Itamar Medical) will be used to examine peripheral vasoconstriction along with microvascular function. The Endo-PAT2000 employs a volume plethysmography approach to quantify peripheral blood volume fluctuations by measuring the finger pulse volume amplitude (PVA) (35). The reactive hyperemia index (RHI) at rest, which reflects the patient's vascular endothelial function, was measured using the Endo-PAT device. PVA was acquired at rest, and during reactive hyperemia, which was produced by releasing a suprasystolic blood pressure cuff for 5 min. The RHI was calculated by dividing the hyperemic finger's postdeflation to baseline pulse amplitude ratio by the contralateral finger's ratio. In normal conditions, the RHI > 1.67, while in endothelial dysfunction, the RHI ≤ 1.67. Endo-Pat only needs 15 min for the whole process.

### Quality of Life

We will use SF-36 to assess the quality of life. It consists of eight parts: general health, physical functioning, bodily pain, role physical, vitality, social functioning, mental health, and role emotional. SF-36 will be recorded at the start of the research and the third month of follow-up.

### Primary and Secondary Outcomes

Primary outcomes: changes in VO_2_ peak after 12 weeks of exercise training in the three groups (HIIT group, MICT group, and control group), and changes in myocardial fibrosis in the three groups.

Secondary outcomes: changes in ST-2, endothelial function, microvascular obstruction, body fat, BMI, the incidence of arrhythmia, and quality of life in three groups after 3 months.

## Discussion

### Our Hypotheses

(1) Compared with the control group, VO_2_ peak will be increased in both the MICT and HIIT groups. The HIIT group may be more effective than the MICT group.

(2) HIIT may improve myocardial fibrosis in patients with AMI.

(3) Compared with the control group, the quality of life will be significantly improved in the MICT and HIIT groups with higher cardiac ejection fraction, improved vascular endothelial function, and lower body fat.

(4) The incidence of arrhythmia will be relatively low in the MICT and HIIT groups.

Exercise-based CR is recommended as a helpful therapy for patients with MI to achieve a healthy lifestyle, and manage risk factors, to reduce all-cause along with cardiovascular mortality (Class I, Level A). The data of CR on patients with AMI mainly come from the end of the last century; however, the clinical management of AMI has changed in the past 30–40 years. The traditional MICT is generally used as the main method to rehabilitate patients with CHD and halt weight gain. Recent studies have found that HIIT may be more effective at increasing cardiopulmonary fitness, improving vascular function, reducing subcutaneous and abdominal body fat, reducing all-cause and cardiovascular mortality, and cardiovascular morbidity, improving insulin resistance than MICT (20, 21). VO_2_peak is likewise a strong individual predictor of mortality and morbidity. We predict that VO_2_peak will be increased in both the MICT and HIIT groups, and it will be higher in the HIIT group.

After MI, myocardial fibrosis will continue to develop, which can result in malignant arrhythmia and cardiac death. New evidence from epidemiological studies and observations suggests that exercise can improve myocardial fibrosis. There are other studies that show that long-term exercise training with repetitive exposure to prolonged vigorous exercise may increase cardiac fibrosis. Myocardial fibrosis was shown to be highly linked with cumulative exercise dosage (36). The effect of HIIT on myocardial fibrosis is uncertain. Therefore, we assume that HIIT may improve myocardial fibrosis in patients with AMI.

## Ethics Statement

The studies involving human participants were reviewed and approved by the Ethics Committee of Guangdong Provincial People's Hospital [No. GDREC2020208H(R1)]. The patients/participants provided their written informed consent to participate in this study.

## Author Contributions

The research was conceived and designed by HM, LG, QG, and LX. XS, XQ, and XC recruited participants, drafted, and revised the manuscript. WL, XL, and HL made significant contributions to the project's conceptualization and design. HM, XS, and XQ managed the clinical practice organization, and finished the statistical analysis. Each author edited the text critically for significant intellectual content and gave final approval to the published version.

## Funding

This research was supported by the grants from National Natural Science Foundation of China (No. 8160284), Natural Science Foundation of Guangdong Province (Nos. 2019A1515011224, 2021A1515011118, and 2021A1515011781), Start-up Funding of National Natural Science Foundation of China (Nos. 8207120182, 8207050582, 8217142362, and 8197091267), the National Key R&D Program of China (No. 2018YFC2001805), Guangdong Medical Science and Technology Research Fund, China (2019118152336191), Guangzhou Science and Technology Foundation and Application Foundation Research Project (Nos. 202102080368 and 202102080033), Traditional Chinese Medicine Bureau of Guangdong Province (20201008), Leading Medical Talents Project in Guangdong Province (Climbing Plan Special Fund) (No. KJ012019431), and High-level Hospital Construction Project of Guangdong Provincial People's Hospital (Nos. DFJH201811, DFJH201922, DFJH2020003, and DFJH2020029).

## Conflict of Interest

The authors declare that the research was conducted in the absence of any commercial or financial relationships that could be construed as a potential conflict of interest.

## Publisher's Note

All claims expressed in this article are solely those of the authors and do not necessarily represent those of their affiliated organizations, or those of the publisher, the editors and the reviewers. Any product that may be evaluated in this article, or claim that may be made by its manufacturer, is not guaranteed or endorsed by the publisher.
